# High-risk profiles for functional and cognitive decline following COVID-19 confinement in nursing home residents: A multicentre observational study

**DOI:** 10.23938/ASSN.1165

**Published:** 2026-07-24

**Authors:** Isabel San Martín-Erice, Cristina García-Vivar, Sara Furtado-Eraso, Ibai Tamayo-Rodríguez, Leticia San Martín-Rodríguez, Nelia Soto-Ruiz, Paula Escalada-Hernández

**Affiliations:** 1 Public University of Navarre (UPNA) Department of Health Sciences Pamplona Spain; 2 IdiSNA, Navarra Institute for Health Research Pamplona Spain; 3 Navarrabiomed Methodology Unit Pamplona Spain; 4 Research Network on Chronicity, Primary Care and Health Promotion (RICAPPS) Pamplona Spain

**Keywords:** Cognitive Dysfunction, Functional Status, Nursing Diagnosis, Nursing Home Residents, Quarantine, Deterioro Mental, Estado Funcional, Diagnóstico de Enfermería, Residentes de Hogares de Ancianos, Cuarentena

## Abstract

**Background:**

To examine changes in functional and cognitive status among nursing home residents following COVID-19 confinement and to identify personal and clinical resident characteristics associated with greater decline.

**Methods:**

A multicentre retrospective observational study was conducted in nursing homes in Navarra, Spain. Functional and cognitive status were assessed before and after the confinement using the Barthel Index (BI), Mini Cognitive Examination (*MEC*), and Global Deterioration Scale (GDS). Resident characteristics, nursing home characteristics, and the nursing diagnosis *Disrupted Thought Processes* were analysed. Univariate and multivariable linear mixed-effects models were used to identify factors associated with changes in BI, *MEC*, and GDS scores.

**Results:**

The final analytical sample comprised 178 residents. All three outcomes deteriorated significantly following confinement. Mean BI and *MEC* scores decreased by 5.0 and 2.0 points, respectively, whereas mean GDS scores increased by 0.24 points. In multivariable analyses, *Disrupted Thought Processes* was associated with greater cognitive decline measured by *MEC* (B = 1.29; 95% CI: 0.40-2.18; p = 0.005). Difficulties in instrumental activities of daily living were associated with greater deterioration in GDS (B = 0.19; 95% CI: 0.02-0.37; p = 0.030) and BI scores (B = 3.60; 95% CI: 0.01-7.19; p = 0.049). Male sex was associated with greater BI decline (B = 4.07; 95% CI: -7.68 to -0.45; p = 0.028).

**Conclusions:**

COVID-19 confinement associates with functional and cognitive decline among nursing home residents. *Disrupted Thought Processes* may be a useful clinical indicator for identifying residents at increased risk of cognitive deterioration during prolonged isolation.

## RESUMEN

**Fundamento:**

Explorar cambios en el estado funcional y cognitivo de residentes en residencias de mayores durante el confinamiento por COVID-19 e identificar variables relacionadas con un mayor deterioro.

**Metodología:**

Estudio observacional retrospectivo multicéntrico en residencias de mayores de Navarra (España). El estado funcional y cognitivo se evaluó antes y después del confinamiento mediante las escalas Índice de Barthel (IB), Mini Examen Cognoscitivo (MEC) y Escala de Deterioro Global (GDS). Se analizaron las características de residentes y centros, y el diagnóstico enfermero *Procesos de Pensamiento Alterados*. Los factores asociados con cambios en las puntuaciones de IB, MEC y GDS se identificaron mediante regresión lineal multivariante (B; IC95%; p).

**Resultados:**

Se incluyeron 178 residentes. Las tres escalas empeoraron significativamente tras el confinamiento; la puntuación media de IB disminuyó 5,0 puntos, la de MEC 2,0 puntos y la de GDS aumentó 0,24 puntos. El diagnóstico *Procesos de Pensamiento Alterados* se asoció con mayor deterioro cognitivo medido mediante MEC (1,29; 0,40 a 2,18; p=0,005). Las dificultades en las actividades instrumentales de la vida diaria se asociaron con mayor deterioro en puntuaciones de GDS (0,19; 0,02 a 0,37; p=0,030) e IB (3,60; 0,01 a 7,19; p=0,049). El sexo masculino se asoció con un mayor deterioro mediante IB (4,07; -7,68 a -0,45; p=0,028).

**Conclusiones:**

El confinamiento por COVID-19 se asoció con deterioro funcional y cognitivo de los residentes de centros sociosanitarios. El diagnóstico *Procesos de Pensamiento Alterados* puede ser un indicador útil para identificar residentes con mayor riesgo de deterioro cognitivo durante periodos prolongados de aislamiento.

## INTRODUCTION

The COVID-19 pandemic posed an unprecedented challenge to healthcare systems worldwide. Older adults living in nursing homes were particularly affected by the imposed isolation, experiencing considerable psychological and physical distress^1^. The pandemic rapidly evolved into a global public health emergency. In the United States of America, approximately 40% of COVID-19-related deaths occurred in nursing homes, compared with 80% in Canada during the first wave, 47% across 22 European countries, and an estimated 68% in Spain^2^^-^^4^. Advanced age was an independent and major risk factor for severe COVID-19 outcomes and mortality, particularly among nursing home residents. Individuals aged 75 years or older had a 13-fold higher risk of death than those aged 65 years or younger^5^.

Nursing homes became epicentres of infection and mortality, with high transmission rates driven by shared living spaces, residents´ underlying health conditions, and the challenges of implementing infection control measures.

To protect residents and control outbreaks, strict measures were implemented worldwide, including visitor bans, suspension of group activities, and prolonged confinement. Although these interventions were essential for infection control, they also had adverse effects on residents’ physical and cognitive health^6^. The nature and intensity of these measures varied according to national policies and available resources. For example, in the United States, efforts focused on isolating suspected cases and restricting visits, helping reduce transmission but increasing social isolation and emotional distres^7^. In Canada, similar measures successfully controlled outbreaks but were associated with accelerated functional and emotional decline^8^. In Asia, approaches ranged from preventive infection control strategies to more restricted confinement policies that substantially limited residents’ mobility^9^. In Europe, particularly in Spain, strict infection prevention measures were widely implemented in nursing homes^10^.

Despite the widespread impact of the pandemic, relatively little is known about the specific functional and cognitive changes experienced by institutionalized older adults following prolonged confinement or the resident characteristics asso-ciated with greater deterioration. One underexplored factor is the nursing diagnosis *Disrupted Thought Processes* (00493), included in the NANDA International (NANDA-I) Nursing Diagnoses classification and defined as a disturbance in cognition, reasoning and, problem-solving^11^. NANDA-I provides a standardized language that enables nurses to identify and communicate health problems within the scope of nursing practice^11^. This diagnosis may help identify residents with underlying cognitive vulnerability and support the development of individualized care plans and interventions aimed at preserving autonomy and safety. Early identification may be particularly important during prolonged periods of isolation, when residents are at increased risk of functional and cognitive decline.

Future public health emergencies remain a significant global concern. It is therefore essential to understand the unintended consequences of protective measures and to identify factors associated with greater vulnerability among nursing home residents in order to improve preparedness strategies.

This study aimed to examine changes in functional and cognitive status among nursing home residents following COVID-19 confinement and to identify resident characteristics associated with a greater decline. In addition, we aim to explore the association between the nursing diagnosis *Disrupted Thought Processes* and the degree of functional and cognitive deterioration observed in this population.

## MATERIAL AND METHODS

### Study design and participants

This multicentre retrospective observational study was conducted in accordance with the Strengthening the Reporting of Observational Studies in Epidemiology (STROBE) guidelines to ensure transparent and rigorous reporting of observational research^12^. The study population comprised residents from seven nursing homes in Navarra, Spain, during the first wave of the COVID-19 pandemic (March-August 2020). Data were collected over a 13 month-period between February 2021 and March 2022.

Eligible participants were aged 65 years or older and had a baseline score of < 4 on the Global Deterioration Scale (GDS)^11^. This threshold was selected because individuals with more advanced cognitive impairment could have had difficulty participating reliably in direct interviews owing to impaired communication, severe temporal disorientation, and limited recall of recent events^13^. Residents receiving palliative care for terminal illnesses were excluded. Written informed consent was obtained from all participants or, where appropriate, from their legal representatives.

Participants were recruited using a non-probability convenience sampling strategy based on the accessibility of participating nursing homes and the availability of eligible clinical records. This approach was considered appropriate given the retrospective nature of the study and the need to access available clinical records. All participating facilities were publicly owned and privately managed. Five nursing homes were located in rural areas and two in the urban area of Pamplona, the capital of Navarra, with 200,000 inhabitants. The larger facility -located in the capital- accommodated 537 residents, whereas the smallest -located southern Navarra- housed 44 residents in a municipality of approximately 1,700 inhabitants. The inclusion of centres with diverse geographical and organisational characteristics enabled the study to capture a broad range of social, demographic, and healthcare contexts. Statistical power, a two-sided significance level of 0.05, and a standardised effect size equivalent to Cohen’s *d* of 0.24 or higher were considered, resulting in a required sample size of 178 residents.

### Variables and measurement instruments

Functional and cognitive status before and after COVID-19 confinement were assessed using validated instruments routinely employed in geriatric care:

- Cognitive function was assessed using the *Mini Examen Cognoscitivo* (MEC), the validated Spanish adaptation of the Mini Mental State Examination (MMSE)^14^^,^^15^. The MEC evaluates five cognitive domains: orientation, registration, attention and calculation, recall, and language/construction. Scores range from 0 to 35, with scores bellow 24 indicating cognitive impairment compatible with dementia. For the descriptive analysis, participants were classified as having *no dementia* (MEC ≥24) or *dementia* (MEC <24)^16^.
- Global cognitive deterioration was assessed using the Global Deterioration Scale (GDS), which classifies cognitive decline into seven stages ranging from no cognitive impairment (Stage 1) to very severe cognitive impairment (Stage 7). This scale is commonly applied in nursing home settings to assess dementia severity^17^.
- Functional status was assessed using the Spanish version of the Barthel Index (BI)^18^, which measures independence in basic activities of daily living, including feeding, toileting, bathing, mobility, dressing, and bladder/bowel continence. Scores range from 0 (complete dependence) to 100 (complete independence).

The primary outcome variables were changes in MEC, GDS, and BI scores between the pre-confinement assessment (before February 2020) and post-confinement assessment (after February 2021).

Independent variables were obtained from residents’ medical records and a study-specific data collection form. Sociodemographic variables included age, sex, and length of stay in the nursing home. Clinical variables included the medications with potential cognitive effects, psychiatric disorders, neurological disorders or neurodegenerative diseases, and previous COVID-19 infection. The principal investigator, a registered nurse, also conducted face-to-face assessments to evaluate subjective anxiety, independence in instrumental activities of daily living (IADL), and the presence of the nursing diagnosis *Disrupted Thought Processes*. This diagnosis was identified following the confinement period through review of residents´ clinical records and direct clinical assessment. Its defining characteristics were assessed according to the NANDA International (NANDA-I)^11^ Nursing Diagnoses classification. The diagnostic procedure and its validation using latent variable analysis have been described elsewhere^19^.

The assessment of IADL focused on activities that could reasonably be performed within the nursing home setting, including managing personal schedules, handling money, or using public transport. Residents were considered to have impaired IADL if they experienced difficulties in at least two of these activities.

Facility-level variables were provided by nursing home managers and included resident-to-nurse ratios during the morning, afternoon, and night shifts reflecting structural staffing levels per shift and not direct one-to-one care delivery; facility size (categorized by number of residents, from <35 to >100); geographical location (urban or rural); and the infection-control measures implemented during the pandemic. These measures included visitor restrictions, universal resident isolation irrespective of infection status, restricted access to communal areas, and suspension of rehabilitation and leisure activities, including occupational therapy, physiotherapy, speech therapy, and recreational programmes.

### Ethical considerations

The study was conducted in accordance with the principles of the Declaration of Helsinki. Participants received detailed information regarding the study objectives, potential benefits, and possible risks before providing written informed consent. Participation was voluntary, and participants retained the right to withdraw at any time. All data were handled confidentially and processed in accordance with applicable data protection legislation. The study was approved by the Clinical Research Ethics Committee of Navarra (reference PI_2020/151).

### Statistical analyses

Participant characteristics, nursing home characteristics, and cognitive and functional assessment scores are presented as means and standard deviations (SDs) for quantitative variables and as frequencies and percentages for qualitative variables.

Changes in MEC, GDS, and BI scores between the pre- and post-confinement assessments were analysed using paired Student’s t-tests for quantitative variables and McNemar´s test for qualitative variables.

Factors associated with changes in BI, MEC, and GDS scores (continuous outcomes) were examined using linear mixed-effects models. Separate univariate models were first fitted for each explanatory variable. Sociodemographic characteristics, clinical variables, nursing home characteristics, and confinement-related measures were entered as fixed effects. Variables associated with functional or cognitive change at *p* <0.05 in the univariate analyses were subsequently included in multivariable models to estimate their independent associations with each outcome. Random intercepts were included to account for clustering residents within nursing homes. Results are reported as unstandardized regression coefficients (B) with corresponding 95% confidence intervals (95% CI). Random-effect parameters are presented as residual variance (σ²), between-centre variance (τ00) and, where applicable, the intraclass correlation coefficient (ICC). All statistical analyses were performed using R statistical software.

## RESULTS

A total of 182 residents were recruited from the seven participating nursing homes. Four records were excluded because of incomplete or inconsistent data, resulting in a final analytical sample of 178 residents. The mean age of participants was 84 years; 62% female. Previous COVID-19 infection was documented in 71% of residents, while 70% were receiving medications with potential effects on the central nervous system. Psychiatric disorders were present in 33% of participants and neurological disorders in 15%. Overall, the study population showed a high prevalence of cognitive, psychological, and functional vulnerability at baseline.

The nursing diagnosis *Disrupted Thought Processes* was identified in 28% of residents and was included as a clinical variable in the multivariable analyses. Detailed sociodemographic and clinical characteristics are shown in Table 1.

**Table 1:** Characteristics of the sample (residents and centres) before the pandemic

Characteristic of n=178 residents	n (%)
Age (years), *mean (SD)*	84 ± 8
Sex (men)	67 (38)
*Length of stay*	
<1 year	0 (0)
1-5 years	115 (65)
>5 years	63 (35)
Time in center (years), *mean (SD)*	5.6 ± 5.2
Difficulties in IADLs	64 (36)
Anxiety	80 (45)
Neurological disease^1^	27 (15)
CNS drug treatment^1^	124 (70)
Psychiatric disease^1^	58 (33)
Nursing diagnosis *Disrupted Thought Processe*s	49 (28)
MEC scale score preCOVID, *mean (SD)*	28.0 ± 5.2
Barthel Index score preCOVID, *mean (SD)*	86 ± 20
GDS scale score preCOVID, *mean (SD)*	1.59 ± 0.89
*Characteristics of the n=7 centres*	*n (%)*
Urban	2 (29)
*Size center (residents)*	
<50	1 (14)
51-100	4 (57)
>100	2 (29)
Ban visits	5 (71)
Isolation for all patients	4 (57)
Common places only for patients without risk contact	5 (71)
Suspension of all activities	4 (57)
*Patients per nurse*	
morning	
0-50	2 (29)
51-100	5 (71)
evening	
0-50	1 (14)
51-100	5 (71)
101-250	0 (0)
>251	1 (14)
nights	
0-50	1 (14)
51-100	1 (14)
101-150	1 (14)
151-200	0 (0)
201-250	1 (14)
>251	3 (43)

SD: standard deviation; CNS: central nervous system; GDS: Global Deterioration Scale; IADLs: instrumental activities of daily living; MEC: Mini Cognitive Exam.

Characteristics of the participating nursing homes and the confinement measures implemented are also summarised in Table 1. Most facilities (71%) were located in rural areas, and more than half accommodated between 51 and 100 residents. Visitor restrictions and limited access to communal areas were implemented in most centres, while over half introduced universal resident isolation and suspended rehabilitation and group activities. Staffing levels varied across facilities, with resident-to-nurse ratios generally higher during the afternoon and night shifts than during the morning shift. In some centres, a single nurse was responsible for more than 250 residents during the night shift. Percentages represent the marginal distribution of each centre-level characteristic and should not be interpreted as indicating the same centres shared all characteristics in the proportions shown.

### Changes in cognitive and functional status following COVID-19 confinement

**Figure 1:**
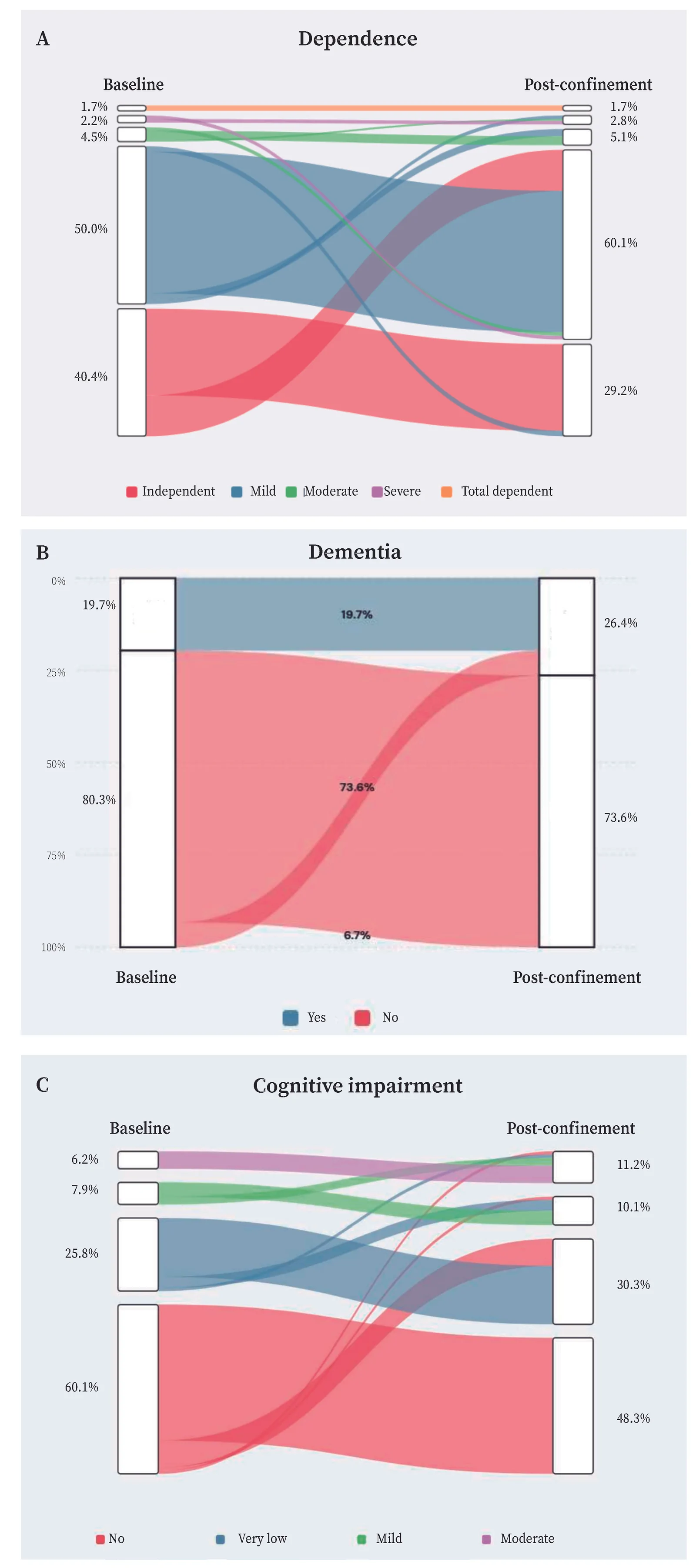
Changes in functional dependence (Barthel Index) (**A**), dementia status (Mini Cognitive Exam) (**B**) and global deterioration (GDS, Global Deterioration Scale) (**C**) before and after confinement measures due to the COVID-19 pandemic. The thickness of the flows indicates the number of participants who followed each trajectory.

Significant deterioration was observed across all three outcome measures following the confinement period. The mean BI score decreased by 5.0 points (p < 0.001), indicating reduced functional independence. The proportion of independent residents decreased from 41.8 to 29.1%, whereas the proportion with mild dependence increased from 50.5 to 58.8% (Figure 1A).

Mean MEC scores decreased by 2.0 points (p < 0.001). The proportion of residents without cognitive impairment declined from 60.2 before confinement to 48.3% afterwards, while the proportion with very mild cognitive impairment increased from 25.8 to 30.3%. Overall, 6.7% of residents progressed from normal cognitive function to dementia during the study period (Figure 1B).

Cognitive deterioration, assessed using the GDS, also increased significantly, with a mean rise of 0.24 points (p < 0.001). The proportion of residents with mild cognitive deterioration increased from 7.9% to 10.1%, and those with moderate deterioration increased from 6.2% to 11.2% (Figure 1C).

### Factors associated with functional and cognitive decline

Univariate linear mixed-effects analyses identified several resident characteristics linked to changes in functional and cognitive outcomes (Table 2). Increasing age was associated with greater functional decline, whereas male sex was associated with smaller reductions in BI scores. Difficulties in IADL were significantly related to greater functional decline and cognitive deterioration, as assessed by the GDS. The nursing diagnosis *Disrupted Thought Processes* emerged as a significantly predictor of changes in all three outcome measures, indicating greater deterioration among residents with this diagnosis. Resident-to-nurse ratio was not significantly related to changes in *MEC* or BI scores. However, the direction of the regression coefficients suggested a tendency towards smaller declines in facilities with higher morning shift staffing levels.

**Table 2:** Factors associated with changes in Barthel Index, MEC, and GDS scores: univariate linear mixed-effects models

Variable	BI	p	MEC	p	GDS	p
	B (95% CI)		B (95% CI)		B (95% CI)	
Age	0.25	0.026	0.03	0.260	0.01	0.012
	(0.03 to 0.47)		(-0.02 to 0.08)		(0.00 to 0.02)	
Men	-4.52	0.013	0.14	0.740	-0.03	0.738
	(-8.07 to -0.97)		(-0.68 to 0.96)		(-0.20 to 0.14)	
Time in center, years	0.03	0.866	0.00	0.901	0.01	0.451
	(-0.31 to 0.37)		(-0.07 to 0.08)		(-0.01 to 0.02)	
Difficulties in IADLs	4.82	0.008	0.63	0.133	0.21	0.014
	(1.25 to 8.40)		(-0.19 to 1.45)		(0.04 to 0.37)	
Subjective anxiety	0.90	0.615	0.57	0.161	0.13	0.117
	(-2.62 to 4.41)		(-0.23 to 1.36)		(-0.03 to 0.29)	
Neurological Disease	2.68	0.278	0.71	0.204	0.20	0.086
	(-2.18 to 7.54)		(-0.39 to 1.81)		(-0.03 to 0.42)	
CNS Drugs	0.59	0.759	-0.07	0.874	-0.05	0.560
	(-3.21 to 4.40)		(-0.93 to 0.79)		(-0.23 to 0.12)	
Psychiatric Disease	0.40	0.832	-0.24	0.578	-0.15	0.078
	(-3.33 to 4.13)		(-1.08 to 0.61)		(-0.32 to 0.02)	
Nursing diagnosis *Disrupted Thought Processes*	3.95	0.046	1.40	0.002	0.23	0.012
	(0.08 to 7.82)		(0.53 to 2.26)		(0.05 to 0.41)	
COVID-19 previous infection	-0.12	0.953	-0.46	0.306	0.04	0.689
	(-3.98 to 3.75)		(-1.33 to 0.42)		(-0.14 to 0.22)	
Ban visits	-6.09	0.073	-0.01	0.988	0.18	0.259
	(-12.75 to 0.57)		(-1.54 to 1.51)		(-0.13 to 0.49)	
Isolation for all residents	-4.54	0.154	0.21	0.776	0.19	0.195
	(-10.80 to 1.72)		(-1.22 to 1.63)		(-0.10 to 0.48)	
Common places only for residents without risk contact	2.50	0.196	0.26	0.558	-0.05	0.589
	(-1.30 to 6.31)		(-0.61 to 1.12)		(-0.23 to 0.13)	
Suspension of all activities	0.82	0.689	0.41	0.382	0.14	0.141
	(-3.23 to 4.88)		(-0.51 to 1.33)		(-0.05 to 0.33)	
Size Center (51-100)	-7.78	0.127	0.19	0.870	0.05	0.846
	(-17.81 to 2.25)		(-2.11 to 2.49)		(-0.42 to 0.51)	
Size Center (>100)	-9.41	0.056	-0.09	0.938	0.09	0.697
	(-19.08 to 0.26)		(-2.30 to 2.13)		(-0.36 to 0.54)	
Urban	-0.08	0.964	0.02	0.952	0.09	0.295
	(-3.59 to 3.43)		(-0.77 to 0.82)		(-0.08 to 0.25)	
Residents per nurse, morning (51-100)	-5.36	0.210	0.38	0.695	0.12	0.538
	(-13.76 to 3.04)		(-1.53 to 2.29)		(-0.27 to 0.51)	
Residents per nurse, evening (51-100)	5.26	0.529	1.51	0.431	0.25	0.527
	(-11.20 to 21.73)		(-2.26 to 5.27)		(-0.52 to 1.02)	
Residents per nurse, evening (>251)	14.17	0.141	1.50	0.495	0.17	0.710
	(-4.74 to 33.07)		(-2.83 to 5.83)		(-0.72 to 1.05)	
Residents per nurse, nights (51-100)	8.57	0.365	1.50	0.488	-0.00	1.000
	(-10.07 to 27.21)		(-2.76 to 5.76)		(-0.87 to 0.87)	
Residents per nurse, nights (101-150)	2.26	0.793	1.05	0.594	0.16	0.686
	(-14.70 to 19.22)		(-2.83 to 4.92)		(-0.63 to 0.95)	
Residents per nurse, nights (201-250)	5.59	0.508	1.53	0.427	0.29	0.463
	(-11.03 to 22.20)		(-2.26 to 5.33)		(-0.48 to 1.06)	
Residents per nurse, nights (>251)	7.39	0.387	1.77	0.363	0.25	0.528
	(-9.42 to 24.19)		(-2.07 to 5.61)		(-0.53 to 1.03)	

CI: confidence interval; BI: Barthel Index; CNS: central nervous system; GDS: Global Deterioration Scale; IADLs: instrumental activities of daily living; MEC: Mini Cognitive Exam, the Spanish version for Mini Mental State Examination (MMSE).

After adjustment for potential confounding variables, impaired IADL and the nursing diagnosis *Disrupted Thought Processes* remained independently associated with changes in cognitive and functional outcomes. Residents with impaired IADL experienced greater functional decline, reflected by larger reductions in BI scores and greater cognitive deterioration. The nursing diagnosis *Disrupted Thought Processes* remained independently associated with greater cognitive decline measured using the MEC (Table 3). Male sex was also independently associated with smaller reductions in BI scores, indicating less functional decline during the confinement period.

**Table 3:** Multivariate multiple regression model for the change in GDS, MEC and BI scores (n=178)

Predictors	B (95%CI)	p	Random effects	
*Change in GDS score*				
(Intercept)	-0.74 (-1.65 - 0.16)	0.107	σ^2^	0.26
Age (years)	0.01 (-0.00 - 0.02)	0.062	τ_00 centre_	0.04
Sex (male)	0.02 (-0.14 - 0.19)	0.775	ICC	0.13
Difficulties in IADLs	0.19 (0.02 - 0.37)	0.030	N _centre_	7
Nursing diagnosis *Disrupted Thought Processes*	0.17 (-0.01 - 0.36)	0.058		
*Change in MEC*				
(Intercept)	-0.52 (-4.92 - 3.88)	0.815		
Age (years)	0.02 (-0.03 - 0.07)	0.370	σ^2^	6.84
Sex (male)	0.12 (-0.71 - 0.96)	0.773	τ_00 centre_	0.00
Difficulties in IADLs	0.38 (-0.45 - 1.21)	0.366	N _centre_	7
Nursing diagnosis *Disrupted Thought Processes*	1.29 (0.40 - 2.18)	0.005		
*Change in BI*				
(Intercept)	-7.31 (-26.37 - 11.75)	0.450		
Age (years)	0.14 (-0.08 - 0.37)	0.207	σ^2^	128.41
Sex (male)	-4.07 (-7.68 - -0.45)	0.028	τ_00 centre_	0.00
Difficulties in IADLs	3.60 (0.01 - 7.19)	0.049	N _centre_	7
Nursing diagnosis *Disrupted Thought Processes*	3.69,(-0.16 - 7.54)	0.060		

GDS: Global Deterioration Scale; MEC: Mini Cognitive Exam. Spanish version for Mini Mental State Examination (MMSE); BI: Barthel Index; IADLs: Instrumental Activities of Daily Living; σ2: random-effect parameters including residual variance; τ00 centre: between-centre variance of the random intercept; ICC (intraclass correlation coefficient): proportion of total variance attributable to differences between centres; N centre: number of nursing homes included in the model.

**Figure 2:**
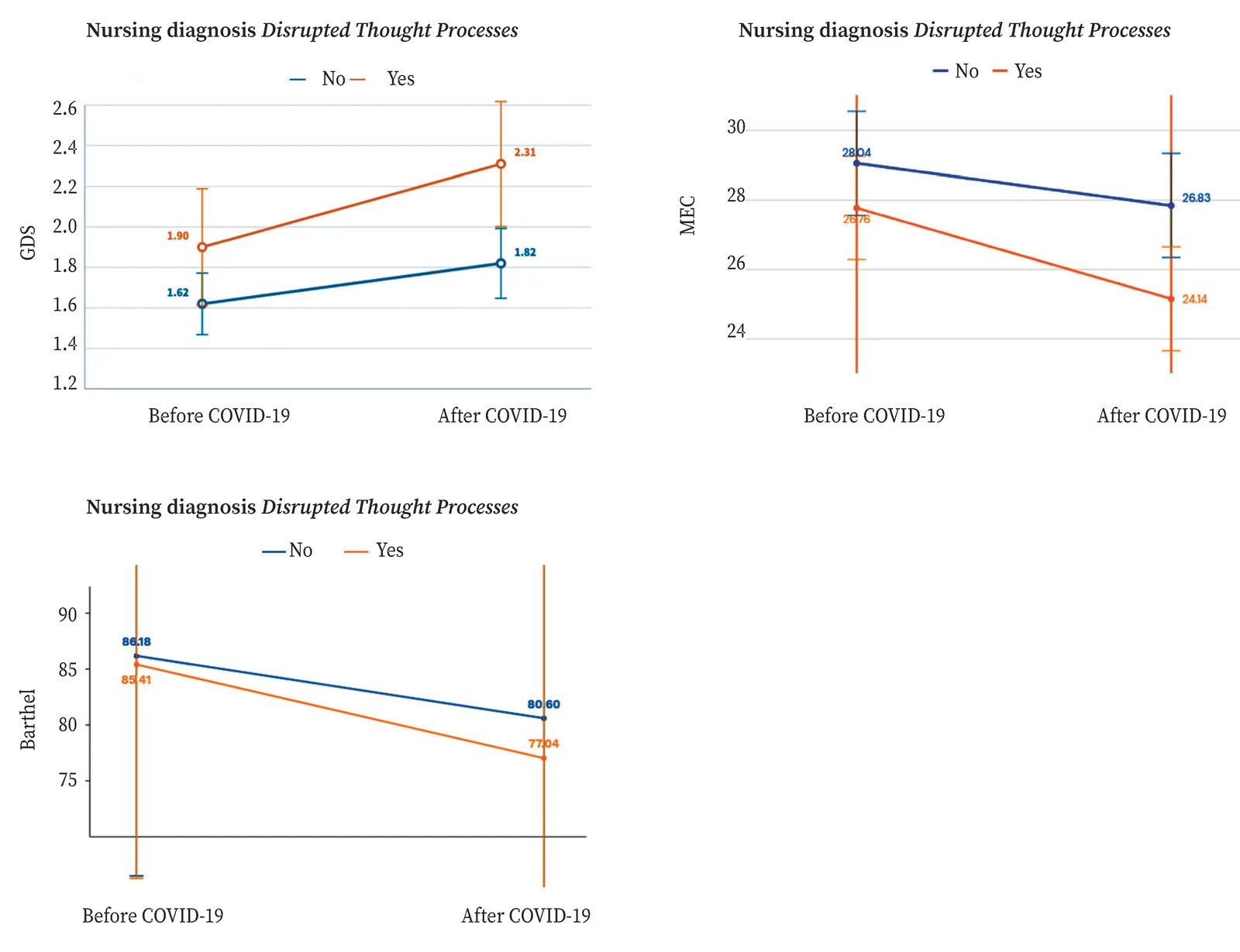
Main effect plot of the presence of the nursing diagnosis *Disrupted Thought Processes* on the evolution of the score changes across Barthel Index, Global Deterioration Scale, and Mini Cognitive Exam (the Spanish version for Mini Mental State Examination / MMSE).

Residents with the nursing diagnosis *Disrupted Thought Processes* experienced greater deterioration in functional and cognitive status during the confinement period than residents without the diagnosis. At baseline, residents with *Disrupted Thought Processes* had poorer cognitive performance, reflected by higher GDS scores and lower MEC scores. In contrast, baseline BI scores were similar between the two groups. Following confinement, residents with the nursing diagnosis showed greater deterioration across all three outcomes measures, indicating more pronounced progression of cognitive and functional impairment (Figure 2).

## DISCUSSION

This multicentre observational study found that COVID-19 confinement associates with significant functional and cognitive decline among nursing home residents. Deterioration is observed across all three outcome measures. Overall, resident--related clinical characteristics appear to explain more of the variability in these changes than facility-level characteristics or specific confinement measures. These findings are consistent with previous studies reporting substantial functional and cognitive deterioration among institutionalised older adults in nursing homes during the COVID-19 pandemic^20^^,^^21^.

A key contribution of this study is the evaluation of the nursing diagnosis *Disrupted Thought Processes* as a potential clinical indicator of vulnerability to decline during prolonged confinement. Residents with this diagnosis experience greater cognitive deterioration and show a consistent tendency towards greater functional decline than those without the diagnosis.

The two-point reduction observed in MEC scores reflects clinically relevant deterioration in cognitive function, affecting domains such as memory, orientation, and reasoning. This finding aligns with the decline (2.5 points) reported by Pérez-Rodríguez *et al*. in a similar population^20^. Likewise, Sepúlveda-Loyola *et al*. associated increased anxiety, depression, sleep disturbances, and reduced physical activity with accelerated cognitive decline in institutionalised older adults, changes that typically evolve more slowly in the absence of pandemic-related stressors^10^. The 0.24-point increase in GDS further supports progression of cognitive impairment following confinement. Although the mean was modest, even small changes in dementia severity may translate into greater dependency, increased care needs, and reduced quality of life. Functional status also worsened significantly, with a mean reduction of five points in BI scores. This decline indicates reduced independence in basic activities of daily living, including dressing, feeding, and mobility, thereby increasing care demands. Reduced opportunities for physical activity, suspension of rehabilitation programmes, and prolonged room confinement probably contributes to physical deconditioning and loss of functional capacity. Similar reductions have been published by Cortés-Zamora *et al*. who reported a five-point BI reduction in 47% of residents^6^, while Pérez-Rodríguez *et al*. found a decrease from 45 to 35 points following lockdown^20^. Importantly, these changes occurred irrespectively of previous COVID-19 infection, supporting previous evidence^22^.

No statistically significant associations are observed between facility-level characteristics, including suspension of therapeutic and recreational routines and visitor restrictions with changes in cognitive or functional outcomes. Nonetheless, previous studies have identified reduced social interaction and reduced cognitive engagement as important factors contributing to decline in institutionalised older adults, regardless of COVID-19 status^23^.

Although resident-to-nurse ratio is not significantly associated with study outcomes, facilities with higher staffing levels during the morning shift tended to show lower declines in MEC and BI. During the pandemic, many nursing homes experienced substantial staffing challenges. Temporary staff often lacked familiarity with residents’ needs, reducing opportunities for personalised care, early recognition of subtle changes in residents’ health, impacting care continuity and quality^24^.

Among resident characteristics, IADL, increasing age, and the nursing diagnosis *Disrupted Thought Processes* associate with greater deterioration*.* Previous studies have reported similar associations between gender and cognitive decline^25^. Female participants - particularly those with limited family contact - experienced greater functional decline that males, possibly due to greater frailty, longer life expectancy, and lower cognitive reserve^26^.

Residents with *Disrupted Thought Processes* experience greater deterioration following confinement than those without the diagnosis. Although this nursing diagnosis has not previously been investigated in relation to prolonged isolation, its defining characteristics reflect disturbances in cognitive processing. Furthermore, impaired IADL has a recognised bidirectional relationship with cognitive decline. Reduced functional autonomy limits participation in cognitively stimulating activities, while progressive cognitive impairment further compromises independence in everyday tasks^25^. These finding suggest that older adults with fragile pre-existing health conditions are particularly vulnerable to the negative effects of prolonged isolation, which limits access to social interactions, physical activities and structured routines, key elements for maintaining mental and physical well-being. Consequently, tailored interventions that prioritize social engagement, cognitive stimulation, and functional support are essential to mitigate these effects in high-risk populations.

This study has several limitations. First, it was conducted in one Spanish region, which may limit the generalizability of the findings to other healthcare systems. Second, participants were recruited using convenience sampling, based on the accessibility of the participating nursing homes and the availability of records, introducing the possibility of selection bias and limiting external validity. Third, although multilevel analyses accounted for clustering within nursing homes, the sample size calculation between centre variability and the study may therefore have been underpowered to detect institutional effects. Fourth, although there is some variability in the time between baseline and follow-up assessments, this interval was relatively homogeneous across residents and therefore was not included as a covariate in the analyses. Nevertheless, this should be considered a potential limitation. In addition, variability in evaluators across centres and assessment periods may have introduced measurement variability, particularly in cognitive and functional assessments. Finally, psychological variables such as depression and anxiety were not assessed, which are closely linked to isolation and may influence cognitive and functional decline. This may have limited the ability to fully analyse the interaction between emotional well-being and functional and cognitive outcomes in institutionalized older adults.

In conclusion, COVID-19 confinement associates with significant deterioration in functional and cognitive performance of nursing home residents. The decline was not uniform across the study population, with residents presenting the nursing diagnosis *Disrupted Thought Processes* showing greater cognitive deterioration and functional decline.

These findings underline the importance of ensuring that infection-control measures in nursing homes should be accompanied by strategies that preserve mobility, autonomy, cognitive stimulation, and continuity of care among institutionalized older adults. From a clinical perspective, residents with pre-existing cognitive vulnerability should receive targeted interventions aimed at maintaining daily routines, encouraging safe mobility, supporting independence in both basic and instrumental activities of daily living, and providing cognitive stimulation tailored to their individual needs.

The nursing diagnosis *Disrupted Thought Processes* may represent a useful clinical indicator for identifying residents at increased risk of functional and cognitive decline during periods of prolonged confinement. Incorporating this diagnosis into comprehensive geriatric assessment may facilitate earlier risk stratification and support the development of individualized nursing care plans. These findings support the clinical value of integrating standardized nursing taxonomies into long-term care settings to identify high-risk profiles and guide individualized care to improve clinical surveillance, continuity of care, and decision-making during future health emergencies.

## Data Availability

We are unable to make the data publicly available as we did not obtain consent from the study participants for open-source sharing during data collection. Nevertheless, the corresponding author can be contacted if someone reasonably requests the raw data from the present study.
